# VTE Assessment for Psychiatric Inpatients–Quality Improvement Project on Acute Male Ward

**DOI:** 10.1192/bjo.2026.11390

**Published:** 2026-06-30

**Authors:** Ruby Griffin

**Affiliations:** South London and Maudsley, London, United Kingdom

## Abstract

**Aims:**

There is an observed poor compliance with assessing venous thromboembolism (VTE) risk when patients are admitted to an acute male ward in Lambeth Hospital. NICE guidelines state that psychiatric patients should have this reviewed at least by first consultant review.

Psychiatric patients have unique risk factors for VTE – antipsychotic use, poor hydration/nutrition and possible use of physical restraint. It is therefore important to assess and treat risk as advised to minimise VTE occurrence. Through improving engagement with risk assessment tool, and documenting if patients are at risk, this will help identify patients who require prophylactic treatment.

**Methods:**

Use electronic patient records (ePJS) to access ward patients’ medical records, every 2 weeks. For each patient carry out the following 1. Establish admission date. 2. Search 'VTE' 'vtrap' 'dvt' on notes. 3. Access VTRAP tool under assessment. Document if completedvs not completed. Change implemented through including VTE assessment in ward round proforma and creation of posters reminding resident (clerking) doctors to assess VTE risks.

**Results:**

18 bed male ward, with average length of stay 32. Baseline data showed 12% completion rate of VTE risk assessment prior to change implemented.

Following change (2 changes implicated of poster + VTE included in ward round proforma), every data collection was over 66%, with all but one being over 80%.

**Conclusion:**

Causes for low compliance for VTE assessment of psychiatry inpatients include lack of knowledge of medical staff that this is a guideline/expectation when admitting patients and lack of knowledge of increased risk in psychiatric patients, and hence need for VTE risk assessment. Through education and engagement, this change has been effective in improving compliance of VTE screening. Improving assessment and education will hopefully in turn improve adherence to VTE prophylaxis and encouragement in improving dynamic risk factors (dehydration/immobility) by the whole MDT.

